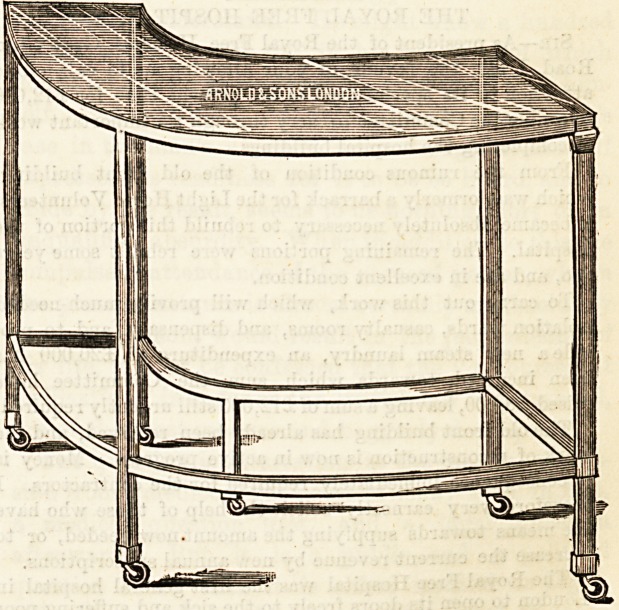# Instrument Table

**Published:** 1893-10-14

**Authors:** 


### PRACTICAL DEPARTMENTS.

### INSTRUMENT TABLE.

A former illustration showed a dressing waggon, made by Messrs. Arnold and Son, Smithfield, in which the shelves are of plate glass. The drawing we give below, also by kind permission of the same maker, represents a curved instrument table, made on a similar plan. The table is curved to surround the operator, and the framework, and legs, tie-rods, &c., are of solid polished brass. The slab is of half-inch polished glass. The table moves lightly and easily on good castors, and in shape and construction will prove of real value to surgeons.

## Figures and Tables

**Figure f1:**